# Japanese parturient body mass index and the role in initial nerve block height of women undergoing cesarean delivery with spinal anesthesia

**DOI:** 10.1097/MD.0000000000020584

**Published:** 2020-06-05

**Authors:** Futaba Miyoshi, Risa Fukushima, Sumire Yokokawa, Shiori Sakuma, Minoru Nomura, Hiroyuki Kinoshita

**Affiliations:** a Department of Anesthesiology, Tokyo Women's Medical University School of Medicine, Shinjuku-ku, Tokyo; b Department of Anesthesiology, Moriya Daiichi General Hospital, Moriya, Ibaraki; c Department of Anesthesiology, Institute of Biomedical Sciences, Tokushima University Graduate School, Tokushima, Tokushima, Japan.

**Keywords:** body mass index, cesarean delivery, gestational age, spinal anesthesia, underweight fetus

## Abstract

The present retrospective cohort study was designed to determine body mass index (BMI) at the delivery in women undergoing cesarean section in a Japanese urban area, and whether the nerve block height after spinal anesthesia upon the cesarean delivery relates to the lower maternal BMI, less gestational age, or underweight fetus at birth in the population.

A total of 401 pregnant women undergoing cesarean delivery with spinal anesthesia were evaluated retrospectively. We examined background differences, including BMI at the delivery, gestational age, and fetal birth weight between the cases with and without the adequate initial nerve block height less than the sixth thoracic vertebral level (Th6) after the spinal dose administration.

The data demonstrated advanced maternal age pregnancy (median 35.5 years) and normal BMI (median 24.7) at the delivery in the population. The patients with the inadequate initial nerve block height immediately after the spinal dose administration documented significantly lower block height compared with those with adequate block height (Th8 [n = 55] vs Th4 [n = 346], *P* < 0.001). There was a risk of the low initial block height caused by either preoperative BMI <23, gestational age <37 weeks, or fetal birth weight <2500 g in the population.

In a Japanese urban area, parturient median BMI undergoing cesarean delivery is in the normal range. Such lower BMI, in addition to less gestational age or underweight fetus, seems one of the factors causing the low initial block height upon spinal anesthesia.

## 1 Introduction

Excessive gestational weight gain, in addition to prepregnancy obesity, is a high risk of cesarean delivery.^[1]^ For a reason, pregnant women are encouraged to prevent obesity during the perinatal period for their safer delivery. Japanese parturient demonstrated the lower obesity prevalence compared with that in western countries already in a decade ago.^[2,3]^ Also, Japan Society of Obstetrics and Gynecology and Japan Association of Obstetricians and Gynecologists, jointly published the guideline to avoid overweight pregnant women in 2014, suggesting that some changes in the situation regarding parturient BMI exist in recent 5 years.^[4]^ However, the current range of prepregnancy, as well as preoperative, body mass index (BMI) in women undergoing cesarean delivery has been unclear in Japan, although almost 10% of parturient in the country receives cesarean delivery.^[2]^

The role of BMI in the success of spinal anesthesia is still controversial. Some studies documented that obesity with increased waist circumference relates to a more extensive spread, as well as longer duration of spinal anesthesia in nonpregnant female patients.^[5,6]^ In contrast, others reported that only a minor increase in the nerve block height occurs in obese patients for cesarean delivery.^[7]^ A previous study demonstrated that the low fetus birth weight indicating the preterm delivery is one of the factors associated with the failure of spinal anesthesia in parturient undergoing cesarean section.^[3]^ However, which factor including maternal BMI at the delivery, gestational age, or the fetus birth weight determines the nerve block height immediately after spinal anesthesia upon the cesarean delivery in the population with a lean or standard body type is unknown.

Therefore, the primary outcome of this retrospective cohort study using data in the recent 5 years is to document that prepregnancy, as well as preoperative, BMI in women undergoing cesarean delivery in a Japanese urban area is less than that reported in western countries. The secondary outcome is to determine whether the nerve block height immediately after spinal anesthesia upon the cesarean delivery relates to the lower maternal BMI at the delivery, less gestational age, or underweight fetus at birth in the population and whether it also estimates any difference of patients’ outcome including intravenously supplemented agents used after delivery of a fetus during the operation.

## 2 Methods

The institutional review board of Tokyo Women's Medical University approved this retrospective observational cohort study with a waiver of informed consent (institutional review board No: 4823-R). The protocol was registered at the UMIN Clinical Trials Registry (No: UMIN000034406). All records of pregnant women undergoing cesarean delivery and the neonates between January 2014 and December 2018 in Tokyo Women's Medical University Hospital, Shinjuku-ku, Tokyo, Japan, were reviewed by 2 authors (FM and RF).

### 2.1 Inclusion and exclusion criteria

**Figure 1:**
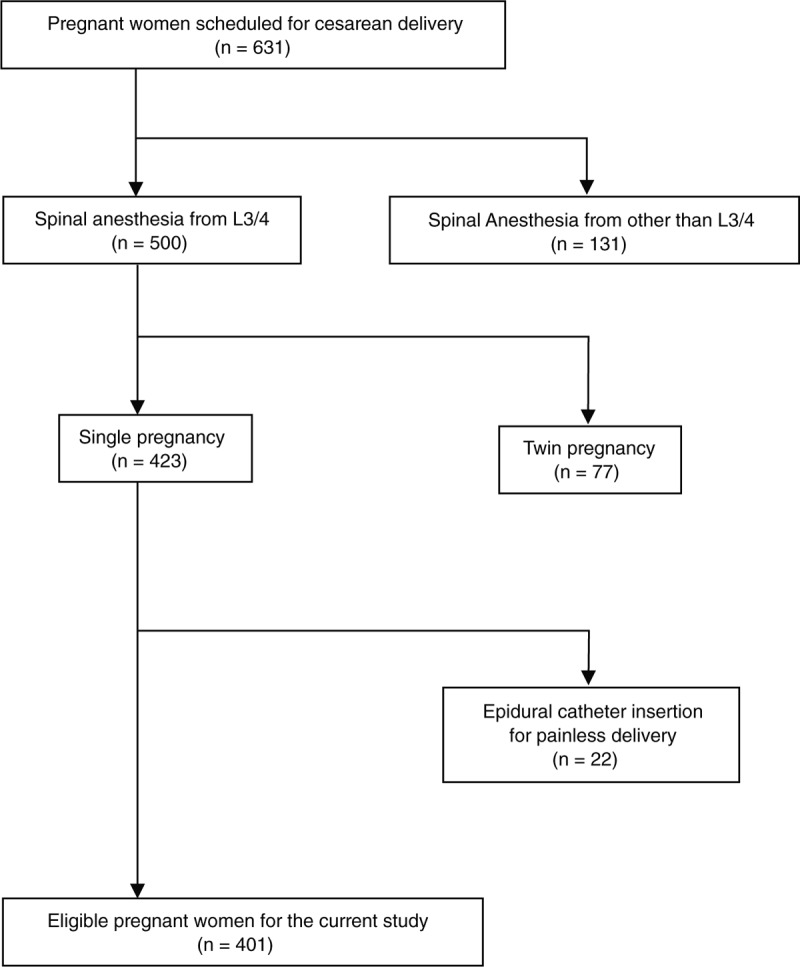
The flow chart indicates the inclusion and exclusion criteria in the present study.

Pregnant women scheduled for cesarean delivery with spinal anesthesia succeeded at the first trial were enrolled (n = 631). Of these, women received spinal anesthesia from L3/4 intervertebral space were only included (n = 500). After that, those with twin pregnancy (n = 77), as well as an indwelled epidural catheter for painless delivery (n = 22), were excluded, and thus, we obtained 401 eligible pregnant women for the present study (Fig. 1).

### 2.2 Anesthetic procedure and data extraction

All eligible patients received our standard doses of 0.5% hyperbaric bupivacaine (9–12 mg) in combination with fentanyl (10–20 μg) intrathecally from L3/4 intervertebral space at the left or right decubitus position. The nerve block height less than the sixth thoracic vertebral level (Th6), or equal and higher than Th6 immediately (3–5 minutes) after the spinal dose administration, was defined as the inadequate and adequate initial sensory block levels, respectively.^[8]^ The highest block height during anesthesia was extracted for each patient. We determined the block height using evaluation of the absent cold sensation serving as a surrogate index for analgesia. The number of cases with supplemented agents including intravenous fentanyl, anesthetics (propofol or midazolam), and anti-inflammatory agents (acetaminophen or flurbiprofen) used after delivery of a fetus during the operation was also collected. We further obtained demographic data, including the age, prepregnancy BMI, BMI at the delivery, gestational age, the prevalence of hypertension disorder of pregnancy, heart disease, and diabetes mellitus for the parturient, and fetal birth weight.

### 2.3 Statistical analysis

Data were presented as mean ± standard deviation (SD), median (interquartile range), or number (%). The power calculation was done using Sample Power 3.0 (IBM Japan Inc., Tokyo, Japan). In the present study, a sample size of 55 gave 100% power to the detected block height difference of 2 vertebral levels at a significance level of 0.05 (SD = 2.1). Statistical analysis using PASW Statistics 18 (IBM Japan Inc., Tokyo, Japan) was performed by unpaired *t* test, Mann-Whitney *U* test, or *χ*^2^ test in combination with risk ratio analysis for parametric variables, nonparametric variables, and the evaluation of frequency, respectively. Differences were considered to be statistically significant when *P* is <.05.

## 3 Results

Table 1 shows the demographics, including maternal age, prepregnancy BMI, BMI at the delivery, gestational age, the prevalence of hypertension, heart disease, and diabetes mellitus, and fetal birth weight in our cohort of 401 Japanese pregnant women in the urban area of Tokyo. The data demonstrated a high rate of advanced maternal age pregnancy (median 35.5 years, 60% [n = 240] of patients was ≥35 years) in the area, and normal BMI (median 24.7) at the delivery, as well as prepregnancy low BMI (median 20.8), although the gestational age (median 38.0 weeks) in the population was at the full term or close to it (Table 1). Of note, a percent of obese pregnant women with the BMI at delivery >30 was only 6.5% (n = 26) in the population, whereas that of normal to underweight parturient was 54% (n = 216). The fetal birth weight was ranged mostly <3000 g (median 2769 g, Table 1).

**Table 1:**
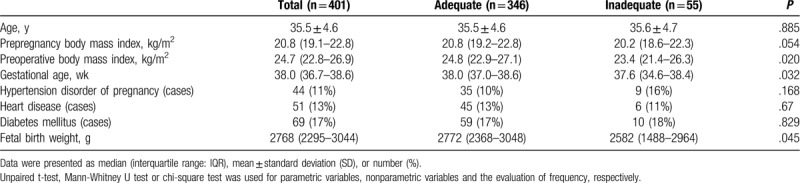
Patient and fetal demographics.

Table 2 demonstrates parameters, which are related to the spinal anesthesia for cesarean section in the present study. The patients with the inadequate initial nerve block height immediately after the spinal dose administration documented about 4 vertebral levels lower block height (Th8) compared with those with adequate block height (Table 2). The trend was the same for the highest block height, although the difference seems to be shrunk to only 2 vertebral levels (Table 2). Intrathecal bupivacaine, as well as fentanyl doses, were not different between the adequate and inadequate initial nerve block height groups, whereas maternal BMI at the delivery, gestational age, and fetal birth weight in the inadequate initial block height group were less than those in the adequate group (Table 1). We further evaluated a risk ratio related to variables including BMI at the delivery, gestational age, and fetal birth weight affecting the inadequate nerve block height immediately after spinal anesthesia using the limits as follows: preoperative BMI <23, gestational age <37 weeks, and fetal birth weight <2500 g (Table 3). We found that each parameter solely added the risk to a chance of the inadequate initial block level in the population of the present study (Table 3). However, the number of cases with supplemented agents, including intravenous fentanyl, anesthetics, and anti-inflammatory agents used after delivery of a fetus during the operation were not different between the inadequate and adequate initial block height groups (Table 2).

**Table 2:**
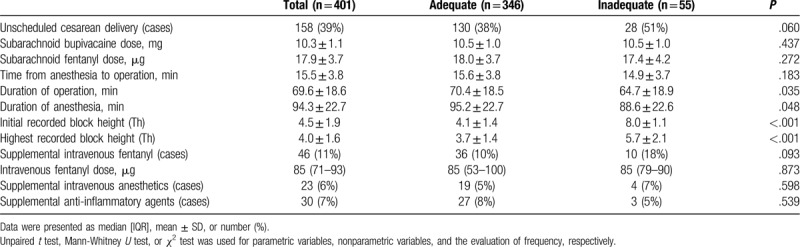
Parameters related to the spinal anesthesia for cesarian section.

**Table 3:**
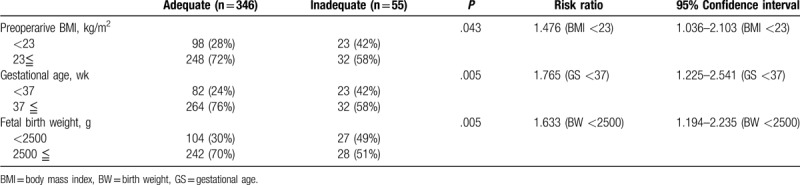
Variables affecting the prevalence of inadequate nerve block height immediately after spinal anesthesia.

## 4 Discussion

The current cohort undergoing cesarean section in a Japanese urban area of Tokyo demonstrated the median maternal age at the delivery was 35.5 years, and 60% of parturient was ≥35 years. Recent large-scale retrospective cohort studies in patients undergoing cesarean delivery in western countries, including the United States, have documented that the advanced age pregnancy with ≥35 years accounts for only <20% of the total parturient in the population.^[3,9]^ A Japanese national survey with the limited cases (n = 915) in a decade ago reported that all maternal age was <36 years in the population.^[2]^ A more recent report, including a total of 97157 pregnant women using the Japan Society of Obstetrics and Gynecology Successive Pregnancy Birth Registry System in 2013, documented the mean maternal age was about 32 years.^[10]^ These results indicate that our cohort shows a high rate of advanced maternal age pregnancy ever reported with higher morbidity of gestational disorders, including hypertension (11%) and diabetes mellitus (17%) compared with that (<10%) in a large-scale report.^[9]^ Therefore, the advanced age in patients undergoing cesarean section seen explicitly only in a Japanese urban area, although whether the condition is currently generalized over the country of Japan is unknown.

We have documented normal BMI at the delivery (median 24.7), as well as prepregnancy low BMI (median 20.8) in the studied population, although the median gestational age was within the full term. A recent large-scale cohort study examining the safety of neuraxial analgesia on pregnant women in the United States contained normal to underweight parturient only <13%,^[9]^ whereas our patients demonstrated the normal to underweight parturient ratio of 54%. These results support the conclusion that preoperative BMI in women undergoing cesarean delivery in a Japanese urban area is currently less than that reported in western countries. The role of the guideline published from Japan Society of Obstetrics and Gynecology and Japan Association of Obstetricians and Gynecologists in 2014 to avoid overweight pregnant women in the BMI of patients undergoing cesarean delivery has been unknown.^[4]^ Therefore, we have hypothesized that some changes in the situation regarding parturient BMI may be evident within the recent 5 years in Japan. In contrast to our thought, the normal to underweight parturient ratio (54%) in our studied population is markedly less than the value (89%) from a large-scale Japanese nationwide search targeted to singleton pregnancies in 2013.^[10]^ The lower percentage of the normal to underweight parturient in the present study is probably due to the differences including the data collection target (ie, patients undergoing cesarean delivery or not) and the ratio of advanced age pregnancy between our population and the previously reported Japanese cohort in 2013. However, we are the first to document the recent percentage of the normal to underweight parturient undergoing cesarean section in Japan, although the regional variation of maternal BMI within the country might exist.

Considering the large population of the normal to underweight parturient undergoing cesarean delivery in Japan compared with western countries, we have further hypothesized whether the lower parturient BMI may affect the nerve block height after spinal anesthesia. Indeed, the role of lower BMI has been unclear in the patients undergoing cesarean section with spinal anesthesia since physician in the western countries does not pay much attention to the population, which is the minority in those areas (<13%).^[9]^ We defined the inadequate nerve block height after spinal anesthesia as less than Th6, according to the criteria in the previous studies,^[8]^ and collected the data regarding nerve block height in the population from the anesthesia records. The patients with the inadequate initial nerve block height immediately after the spinal dose administration documented about four vertebral levels lower block height compared with those with adequate block height. In contrast, the number of cases with supplemented agents, including intravenous fentanyl, anesthetics, and anti-inflammatory agents used after delivery of a fetus during the operation, were not different between the inadequate and adequate initial block height groups. Therefore, the difference in initial nerve block height of 4 vertebral levels may not affect patients’ outcomes during the cesarean delivery. Also, one can argue that the time from intrathecal dose administration to the operation for 15 minutes may be enough to wait for reaching the patient's analgesia level becomes sufficient in the population. However, we do not have appropriate data showing the block height immediately before the operation to support the conclusion. Indeed, the difference of the highest block height was shrunk to only 2 vertebral levels, indicating the possibly delayed effect of spinal anesthesia in patients with the inadequate initial block height.

In the next step, we evaluated which factor, including maternal BMI, gestational age, or the fetus weight, determines the initial nerve block height immediately after spinal anesthesia in our population since these 3 factors appear to affect each other in the maternal evaluation during the perinatal periods. Indeed, values of maternal BMI at the delivery, gestational age, and fetal birth weight in the inadequate initial block height group were less than those in the adequate group. Also, the parameters, including preoperative BMI <23, gestational age <37 weeks, or fetal birth weight <2500 g, solely added the risk to a chance of the inadequate initial block level. A previous study demonstrating that the low fetal birth weight indicating the preterm delivery is one of the factors associated with the failure of spinal anesthesia in parturient undergoing cesarean section supports our conclusion.^[3]^ Collectively, the BMI in combination with gestational age may become valuable surrogate parameters instead of fetal birth weight for anesthesiologists to adjust the intrathecal dose of local anesthetics and opioids before spinal anesthesia for the cesarean section at least in our study population.

Several limitations in this study must be quoted. First, our study was conducted in a retrospective fashion with a specific cohort in a single university hospital, and thus, some limitations caused by the study methodology might have occurred. Second, all patients received our standard intrathecal doses of hyperbaric bupivacaine in combination with fentanyl from the same intervertebral space. However, the dose varied, and injection was done at the left or right decubitus position depending upon each patient favorite, indicating that such procedures may affect the nerve block height. Third, supplemented agents after delivery of a fetus were administered dependently upon the decision of an anesthesiologist in charge for each cesarean section, and thus, we cannot neglect the possibly prophylactic doses, which may alter our conclusion. Therefore, prospective, multi-hospital data in the same area will require to overcome the disadvantage. Also, we would conduct a future study with the same protocol of spinal anesthesia for every patient, as well as the adjusted criteria to give supplemental agents after delivery of a fetus in parturient undergoing cesarean delivery.

We found that the median BMI at the delivery in women undergoing cesarean section in the Japanese urban area is in the normal range and much less than that reported in western countries. Either the maternal BMI <23 at the delivery, gestational age <37 weeks, or underweight fetus at birth <2500 g in the population is at risk for the inadequate nerve block height less than Th6 immediately after spinal anesthesia upon the cesarean delivery. We recommend anesthesiologists in charge of cesarean delivery to evaluate the maternal BMI, as well as the gestational age before the spinal anesthesia, and to adjust the intrathecal dose of local anesthetics and opioids depending on the values to obtain adequate block height for the surgery promptly.

## Author contributions

FM and RF designed the study, collected the data and prepared the manuscript; HK designed the study, performed the analysis, and prepared the manuscript; SY, SS, and MN perfbrmed the analysis.
